# Machine Learning Modeling for Spatial-Temporal Prediction of Geohazard

**DOI:** 10.3390/s23229262

**Published:** 2023-11-18

**Authors:** Junwei Ma, Jie Dou

**Affiliations:** 1 Badong National Observation and Research Station of Geohazards, China University of Geosciences, Wuhan 430074, China; doujie@cug.edu.cn; 2 Three Gorges Research Center for Geohazards of the Ministry of Education, China University of Geosciences, Wuhan 430074, China

## 1. Introduction

Geohazards, such as landslides, rock avalanches, debris flow, ground fissures, and ground subsidence, pose significant threats to people’s lives and property [1]. Recently, machine learning (ML) has become the predominant approach in geohazard modeling [2,3,4,5,6,7,8,9,10,11,12,13], offering advantages, like an excellent generalization ability and accurately describing complex and nonlinear behaviors. However, the utilization of advanced algorithms in deep learning remains poorly understood in this field [7,8]. Additionally, there are fundamental challenges associated with ML modeling, including input variable selection, uncertainty quantification, and hyperparameter tuning [3,5,6,7,8,9,10,11,12,13].

This Special Issue presents original research exploring new frontiers and challenges in applying ML for the spatial-temporal modeling of geohazards. The topics covered include geohazard modeling, spatial-temporal prediction, ML, deep and reinforcement learning, the metaheuristic optimized ML approach, and physics-based and data-driven hybrid modeling.

## 2. Overview of Contribution

This Special Issue titled “Machine Learning Modeling for Spatial-Temporal Prediction of Geohazard” comprises eleven high-quality papers, including one systematic review article and ten original research articles conducted by researchers from Canada, China, Iran, Malaysia, Pakistan, and Sweden. These ten research articles can be categorized as follows: the susceptibility analysis of glacier debris flow and landslides (contributions 1–3), the displacement prediction of reservoir landslides (contributions 4–6), slope stability prediction and classification (contributions 7–8), building resilience evaluation (contribution 9), and the prediction of rainfall-induced landslide warning signals (contribution 10). Modern ML techniques, including metaheuristic optimized ML, deep learning, and automated ML, have been applied to the spatial-temporal modeling of geohazards in various regions, such as Kurdistan in Iran, Karakorum Highway in Pakistan, and Chongqing, G318 Linzhi Section, and the Three Gorges Reservoir area in China.

Geohazard susceptibility mapping is the central theme of this Special Issue (contributions 1–3). For instance, the susceptibility mapping of glacier debris flows along the G318 Linzhi Section in China was generated using remote sensing imagery and a convolutional-neural-network-based image segmentation model, DeepLabv3+ (contribution 1). In the context of landslide susceptibility mapping, a deep learning model that combines extreme machine learning, a deep belief network, back propagation, and a genetic algorithm has been proposed and validated in Kamyaran in the Kurdistan Province, Iran (contribution 2). The proposed deep learning models achieved satisfactory performances, with values exceeding 0.90 (contributions 1 and 2), underscoring the effectiveness of deep learning in geohazard susceptibility mapping. In the research conducted by Hussain et al. (contribution 3), landslide susceptibility mapping was compared using random forest, extreme gradient boosting, k-nearest neighbor, and naive Bayes in a case study along Karakorum Highway in Northern Pakistan.

Another significant focus of this Special Issue is the prediction of reservoir landslide displacements. Due to seasonal rainfall and periodic reservoir fluctuations, the deformations of reservoir landslide are characterized by a step-like behavior. Innovative approaches based on the decomposition and ensemble principle have been introduced to predict displacements in the cases of the Shiliushubao (contribution 4), Baijiabao (contribution 5), and Baishuihe landslides (contribution 6). These approaches incorporate mode decomposition, input variable selection, individual prediction, and ensemble prediction to achieve a satisfactory performance, nearly optimizing the goodness of fit. Decomposition techniques, such as complete ensemble empirical mode decomposition (contributions 4 and 5) and variational mode decomposition (contribution 6), are utilized to break down cumulative displacement into trend, periodic, and random components. Methods like edit distance for real sequence (contribution 4), gray relational analysis, and association rule mining (contribution 6) have been proposed for the selection of input variables. For individual prediction, various methods, including metaheuristic optimized support vector regression (contribution 4), back propagation neural networks (contribution 6), and gated recurrent unit deep learning (contribution 5), are employed to predict the decomposed displacements, which are then aggregated into a final ensemble prediction. In particular, Zhang et al. (contribution 4) evaluate the performance of hyperparameter tuning using metaheuristic techniques, such as the bat algorithm, grey wolf optimization, dragonfly algorithm, whale optimization algorithm, grasshopper optimization algorithm, and sparrow search algorithm. The abovementioned works (contributions 4–6) contribute significantly to the field of model decomposition, input variable selection, and hyperparameter tuning.

Slope stability prediction and classification (contributions 7 and 8) represent another prominent theme in this Special Issue. Wu et al. (contribution 7) developed a stability prediction model for slope with predetermined shear planes with Box–Jenkins’ modeling approach using a mechanical-informed dataset. For the first time, an automated ML model for slope stability classification has been developed with minimal human intervention by Ma et al. (contribution 8). The AuotML model provides an attractive alternative to traditional ML practice, especially for early-stage researchers with limited expertise in ML.

In the work by Zhang et al. (contribution 9), an ML-based model for assessing the resilience of buildings was developed and evaluated in Banan District, a typical mountainous city in Chongqing, China. Furthermore, Zhang et al. (contribution 10) proposed a hybrid model that combines an attention-based temporal convolutional neural network with entropy weight methods for predicting rainfall-induced landslide warning signals.

Additionally, in a review article entitled “Scientometric Analysis of Artificial Intelligence (AI) for Geohazard Research”, Jiang et al. (contribution 11) conducted a scientometric review of artificial intelligence for geohazard research based on thousands of records from the Web of Science core collection. This analysis identified and visualized the most productive researchers, institutions, and emerging research topics using animated maps, and it also provided recommendations for future directions. This scientometric review holds promise in offering a comprehensive and objective overview of publication characteristics and emerging trends for researchers in the field.

## 3. Conclusions

This Special Issue provides a forum for presenting original research that delves into novel frontiers and confronts challenges in utilizing ML for geohazard susceptibility mapping, geohazard prediction, slope stability prediction, building resilience evaluation, and landslide early warning systems. Within these domains, advanced ML techniques, including deep learning, metaheuristic optimized ML, ensemble learning, and AutoML, have been introduced. We anticipate that these innovative techniques and approaches will be valuable contributions that are warmly received by both researchers and practitioners in the field.
